# Outcomes of Wound Dehiscence after Penetrating Keratoplasty and Lamellar Keratoplasty

**DOI:** 10.1155/2018/1435389

**Published:** 2018-07-08

**Authors:** Xin Wang, Ting Liu, Sai Zhang, Xiaolin Qi, Suxia Li, Weiyun Shi, Hua Gao

**Affiliations:** ^1^Qingdao University, Qingdao 266071, China; ^2^Shandong Eye Hospital, Shandong Eye Institute, Shandong Academy of Medical Sciences, Jinan 250021, China; ^3^Qingdao Eye Hospital, Shandong Eye Institute, Shandong Academy of Medical Sciences, Qingdao 266071, China

## Abstract

**Objective:**

To investigate the incidence, causes, occurrence time, and range of wound and outcomes of wound dehiscence in patients treated by penetrating keratoplasty (PK) or lamellar keratoplasty (LK).

**Methods:**

We retrospectively reviewed medical records of keratoplasty in Shandong Eye Hospital from January 2006 to June 2017. Thirty-one eyes of 30 patients had sustained wound dehiscence (WD) after surgical treatment. The surgical type, causes, occurrence time, extent of the wound, treatment, and outcomes were recorded.

**Results:**

The study population consisted of 26 men and 4 women. The mean age at the occurrence of WD was 44.6 years old (range: 12–78 years), and the mean time from keratoplasty to WD was 45.9 months (range: 1–204 months). WD occurred in 23 eyes (23/1385, 1.66%) after PK and 8 eyes (8/1632, 0.49%) after LK (*p* < 0.05). Twenty-seven eyes (27/31, 87.0%) had trauma-induced dehiscence. The mean range of dehiscence was 5.5 o'clock. The vision ranged from 20/50 to light perception after wound suture. The eyes receiving LK had fewer serious complications than PK.

**Conclusions:**

Compared with LK, PK seems to be more prone to result in wound dehiscence. The WD after LK may be less severe. The visual acuity after treatment of WD can be worse in the eyes with PK than LK.

## 1. Introduction

Corneal diseases represent the second leading cause of blindness globally. Keratoplasty is the major surgical procedure for visual restoration of corneal blindness. Corneal wound dehiscence (WD) is not an uncommon complication after keratoplasty. Although its incidence is relatively low, compared with other complications [1], WD may lead to delayed visual recovery, corneal graft edema, immune rejection, endophthalmitis, suprachoroidal hemorrhage, severe, and even irreversible damage to the vision function [2, 3]. In the recent decade, lamellar keratoplasty (LK) has an increasing trend. Due to retaining of the posterior corneal stroma, it theoretically has better biomechanical stability and might reduce the risk of postoperative WD. In the current study, we retrospectively reviewed medical records of keratoplasty in our hospital and analyzed the patient characteristics, causative factors, clinical features, and outcomes of WD in patients with penetrating keratoplasty (PK) or LK.

## 2. Methods

From January 2016 to June 2017, 3017 keratoplasties were performed in Shandong Eye Hospital, including 1385 PK surgeries, 1632 LK surgeries, and 75 endothelial transplantations. The patients were 1988 males and 1029 females. We retrospectively reviewed the medical records of these patients and recorded the characteristics, risk factors, and outcomes of corneal WD in patients with different surgical approaches. Data collected included patient age and gender, indication for keratoplasty, surgical procedures, duration between keratoplasty and WD, causative events for WD, size of dehiscence, treatment procedures, and vision outcomes after surgical repair.

All patients with WD were given eye shields or glasses to protect the eyes before treated with emergency corneal graft surgery. Patients who did not have eye content exposure or other serious complications underwent original corneal graft repair under topical anesthesia or general anesthesia. In the eyes with iris or vitreous prolapse, corneal graft rejoint surgery was performed, and complicated fundus surgery was combined if needed. WD was sutured using 10-0 nylon sutures. Postoperatively, systemic and intravenous antibiotics were administered, as well as topical antirejection drugs. All data analyses were performed using SPSS statistical software (version 17.0, SPSS, Inc, Chicago, Illinois, USA). Quantitative data are presented as the mean ± standard deviation (range). A value of *p* < 0.05 was considered statistically significant.

## 3. Results

### 3.1. Patient Demography and Indications for Keratoplasty

**Figure 1:**
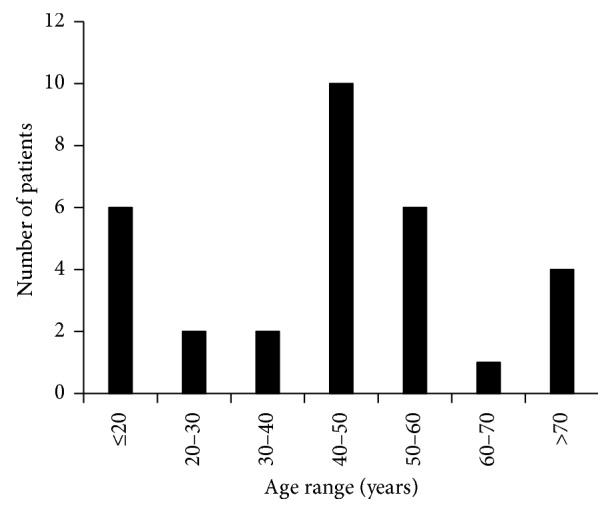
Age of patients with wound dehiscence.

Thirty-one eyes from 30 patients (1.0%) suffered WD after keratoplasty, including 26 males (86.7%) and 4 females (13.3%). The age span of the patients with WD was between 12 and 78 years old with the mean age being 44.6 ± 18.3 years old (Figure 1). The patients included 18 farmers, 4 students, two civil servants, one freelancer, one hobo, and 5 with unknown professions. The follow-up was 1–5 years.

**Figure 2:**
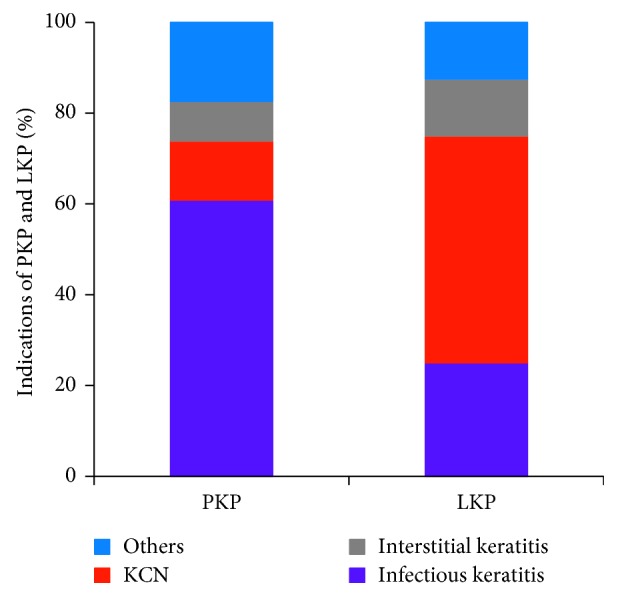
The indications of PK and LK.

**Figure 3:**
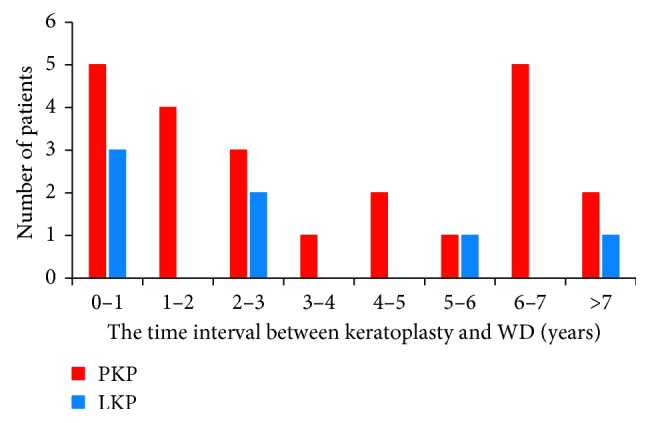
The time interval between keratoplasty and occurrence of WD.

The corneal graft WD occurred in 23 eyes (23/31, 74.2%) after PK and 8 eyes (8/31, 25.1%) after LK. The incidence of WD after PK and LK was 1.66% and 0.49%, respectively (*p*=0.01). The major indications for PK among these patients were fungal keratitis (8 eyes, 34.8%), herpes simplex virus keratitis (5 eyes, 21.7%), keratoconus (3 eyes, 13.0%), and bullous keratopathy (3 eyes, 13.0%). The common indications for LK included keratoconus (4 eyes, 50.0%), fungal keratitis (2 eyes, 25.0%), interstitial keratitis (1 eye, 12.5%), and ocular chemical injury (1 eye, 12.5%) (Figure 2). The mean interval between the initial keratoplasty and occurrence of WD was 45.9 months, with 61.3% within 4 years; the mean interval between the initial PK procedure and WD was 45.0 ± 36.4 months, with 56.5% within 4 years (range, 1 to 126 months); and the mean interval between the initial LK and WD was 48.4 ± 66.2 months, with 75% within 3 years (range 5 to 204 months) (Figure 3).

### 3.2. Causes and Severity of Wound Dehiscence

As shown in Table 1, WD resulted from known trauma in 27 eyes (87.0%), was spontaneous in 4 eyes (13.0%), and had an unknown predisposing cause in 6 eyes (19.4%). The specific trauma treated by PK included strike by obvious objects (41.9%), spontaneous injury (12.9%), strike by no obvious cause (19.4%), hurt by hand or elbow (22.6%), and accidental falling (3.2%). Twenty-two of the 30 patients purchased protective goggles, but none had worn protective goggles when they were injured.

Slit lamp examination showed that the corneal fissure was located in the corneal graft-host interface. Nearly one-third (10/31) of the eyes had sutures in place after trauma. 37.5% of the eyes with LK had sutures, while 30.4% of the eyes with PK had sutures in place. The suture technique used in the keratoplasty was interrupted suture. The mean range of dehiscence was 5.5 o'clock in the eyes with sutures, while 5.2 o'clock in the eyes without sutures. There is no significant difference in wound dehiscence (*p* > 0.05). And there is also no statistical significance (*p* > 0.05) in PK or LK. Two cases were excluded in that we did not know the location of WD. The mean range of dehiscence was 5.7 o'clock in all the eyes, 5.1 o'clock in the eyes with LK, and 5.9 o'clock in the eyes with PK. WD covered 1–3 clock hours in 6 eyes (20.7%), 4–6 clock hours in 11 eyes (37.9%), 7–9 clock hours in 11 eyes (37.9%), and 10–12 clock hours in one eye (3.5%).

Four patients after PK and 2 after LK had wound disruption of 1 to 3 clock hours. Seven after PK and 4 after LK had wound disruption of 4 to 6 clock hours. Nine after PK and 2 after LK had wound disruption of 7 to 9 clock hours. One after PK had disruption of 10–12 clock hours. The wound dehiscence encompassed the inferior temporal quadrant in 4 eyes (26.7%), inferior nasal quadrant in 6 eyes (40.0%), superior nasal quadrant in 9 eyes (60.0%), and superior temporal quadrant in 10 eyes (66.7%). The wound dehiscence with 180° or more occurred in 14 eyes (48.3%) with 12 eyes in PK and 2 eyes in LK. And the incidence of extensive wound dehiscence is not different between PK and LK (*p* > 0.05).

### 3.3. Accompanied Complications

With the increase in the range of corneal WD, the degree of eye prolapses increased. Accompanied complications included iris prolapse in 5 eyes (16.1%), lens expulsion or dislocation in 15 eyes (48.4%), and extrusion of vitreous in 11 eyes (35.5%). In the eyes treated by PK, the complications were iris prolapse in 4 eyes, lens expulsion or dislocation in 15 eyes, and extrusion of vitreous in 10 eyes. In the eyes treated by LK, the complications were iris prolapse in 1 eye and extrusion of vitreous in 1 eye. The lens in one eye and the vitreous in the other eyes were not seen clearly. The extrusion of the lens and vitreous mainly occurred in the patients with an extent of wound disruption ≥ 6 o'clock hours (7/10, 70%).

### 3.4. Therapeutic Outcomes

The duration between the occurrence of corneal graft dehiscence and therapy was 2 to 72 hours. Among 31 eyes of 30 patients, 31 eyes, including 22 eyes after PK and 8 eyes after LK, just had the graft repaired, and only 1 eye after PK was treated with combined anterior chamber angioplasty surgery because of flat anterior chamber.

Final visual acuity was 20/200 or better in 12 eyes (40%), better than hand motions (HM) to 20/200 in 11 eyes (36.7%), HM to light perception (LP) in 7 eyes (23.3%), and unknown in one eye. In the follow-up period, BCVA was improved in 19 eyes (65.5%), including 16 eyes with PK and 3 eyes with LK, unchanged in 9 eyes (31.0%), including 6 eyes with PK and 3 eyes with LK, and decreased in one eye with LK (3.5%). Patients after treatment of LK achieved better final visual acuity than those after PK, but the final visual acuity and the recovery of visual acuity were of no statistical significance (*p* > 0.05). In addition, patients with LK were less likely to suffer lens loss (*p* < 0.05). Although LK patients had less extrusion of the lens (*p* > 0.05) and vitreous (*p* > 0.05), there was no significant difference. Furthermore, there was no difference in the range of WD between PK and LK (*p* > 0.05).

## 4. Discussion

The cornea never regains the original tensile strength after keratoplasty [4], whether PK or LK. There is a risk of corneal WD in the postoperative cornea. The incidence of WD after corneal transplantation ranges from 0.6% to 5.8% [3, 5, 6], and one major reason is trauma reported to be 1.28%–2.53% [5–8]. In our study, the incidence of WD after keratoplasty was 1.0%, lower than the other reports. This may be because some patients were treated in the local medical units and were not referred to our hospital.

It was reported that the incidence of WD was related to age. Older people were found to be more likely to develop graft WD [5]. In contrast, some researchers believed that young patients with keratoconus were more liable to develop WD [9–11]. In our series, no significant age-related findings were found with 4 (12.9%) patients of less than 18 years old and 6 patients (19.4%) of more than 60 years old. Men were reported to be the majority of the injured patients [5]. Considering the working environment of our patients, farmers and students (61.3%) were easy to be hurt because their protective measures were poorer than other occupations. Wearing protective goggles was very necessary to avoid WD.

WD after keratoplasty has been divided into traumatic WD and spontaneous WD. In our study, the incidence of traumatic WD was 87.1%, but we also needed to notice that there were some cases with no obvious causes. Long-term using of topical corticosteroids could increase the risk of corneal WD after the removal of sutures [7]. The safety and side effects of postoperative glucocorticoid therapy should not be ignored. Since immunosuppressive agents could be partially replaced with topical corticosteroids, the amount of corticosteroids may be reduced and topical immunosuppressive eye drops are administered instead [7].

It was reported that corneal WD mostly occurred within two years. The mean interval between keratoplasty and WD was 45.9 months in our study, and dehiscence occurred during the first 4 years in 61.3% of the eyes. The longest duration between keratoplasty and WD occurrence in China was 9 years [12], and the longest in our survey was 17 years. According to Tran et al., the longest was 20 years [13]. All traumatic WDs were observed to occur in the corneal graft-host interface [3, 14], and our finding is consistent with it. This phenomenon indicated that the tensile strength of corneal graft-host junction after keratoplasty was weaker than original corneal tensile strength. It will never regain the same level of normal intact tissue even in many years. An experimental investigation disclosed that the junction after corneal transplantation could never return to normal intensity. In addition, increasing evidence indicated that WD was a lasting risk in all patients undergoing keratoplasty, regardless of their age, the type of operation, indication for surgery, and time to dehiscence after corneal transplantation [15]. Therefore, such patients should pay attention to take long-term protective measures such as wearing safety glasses.

In our hospital, the sutures were removed within 1.5 years. Therefore, the sutures were in place in the eyes in which WD happened within 1.5 years. To our surprise, we found that WD without sutures did not lead to more extensive WD compared with those with sutures. So we think that the remaining sutures did not affect WD. The extend of graft dehiscence attributed to the trauma after WD. This result was not consistent with other reports. We think the reason may be that our sample is too small. Meyer found that leaving sutures may maintain the integrity of the graft-host junction and dehiscence with sutures led to less dehiscence [16]. Even in the different type of operation, the remaining sutures did not help the eyes to have lower rate of WD. And the rate has no significant difference in LK or PK.

In the current study, the type of operation was found to be an important factor of the occurrence of WD. The incidence of WD was 1.66% after PK and 0.49% after LK (*p* < 0.05). We could get a primary conclusion that LK, compared with PK, was less liable to lead to WD. Retaining the posterior corneal stroma during LK can better recover vision and lower postoperative complications.

WD may result in many serious ocular complications including iris prolapse, crystalline or intraocular lens expulsion or dislocation, and extrusion of vitreous. As previously reported, lens expulsion or dislocation was associated with poor prognosis and the final visual acuity. We noticed that PK patients tended to suffer more severe complications from WD. The reason may be that the cornea still remained a part of the autologous corneal tissue after LK and was protected with the help of full thickness of the Descemet membrane [17, 18].

**Figure 4:**
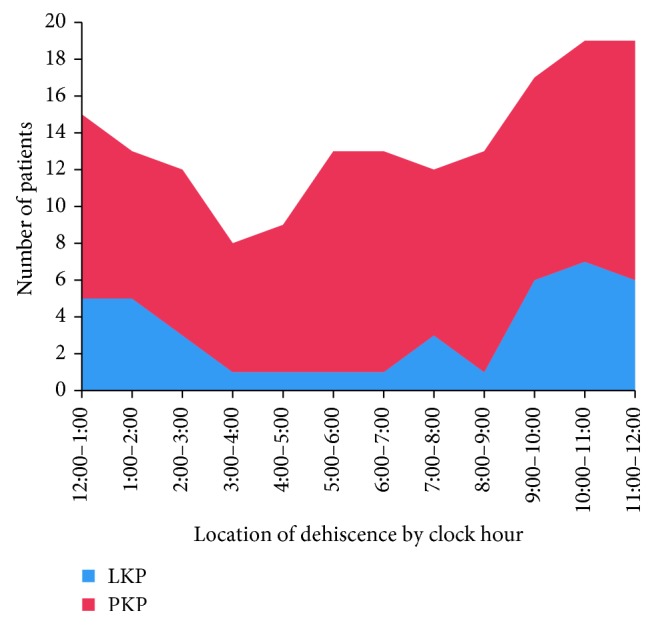
The location of wound dehiscence.

Once WD occurs, the degree of injury would directly affect the patient prognosis. It was reported that in patients with poor prognosis after injury, only 1/3 to 1/2 of patients had visual acuity of 20/200 [19]. In our patients with WD, only 9 had corrected visual acuity of 20/200, and the visual acuity of 11 patients was below FC. With the increase in the range of WD, the visual acuity and prognosis of patients got worse. In this study, WD was more common in the superior quadrant of the graft, especially in the superior temporal quadrant (Figure 4). We think this quarter was prone to occurrence of WD because temporal quadrant is without the help of the bone [18]. Then, the graft was directly faced with trauma. So the incidence of WD may be higher in the superior temporal quadrant. However, our opinion was not consistent with other reports. Farley and Petit reported that most of the eyes had dehiscence in the inferior quadrant because of lack of protection by the nose and eyelids [20]. And there was no quadrant that was prone to WD in previous observations [5, 21]. We also think that the specific trauma may decide the direction and location of the WD.

In conclusion, WD is a risk factor for patients undergoing corneal transplant. Compared with LK, PK seems to be more prone to result in wound dehiscence. The WD after LK may be less severe. The visual acuity after treatment of WD can be worse in the eyes with PK than LK. To reduce the incidence of WD after corneal transplantation, the patient's condition needs to be comprehensively analyzed before selecting appropriate surgical approaches, regular postoperative follow-up is important, and the protective awareness of the patient and family members should be improved.

## Figures and Tables

**Table 1:** Characteristics of wound dehiscence following keratoplasty.

Case	Indication for keratoplasty	Type of keratoplasty	Age at the time of trauma (years)	Cause of trauma	Interval between trauma and keratoplasty (months)	Final visual acuity
1	PBK	PK	40	Unknown	6	FC/BE
2	HSK	PK	51	Struck by iron drill	13	FC/20 cm
3	KCN	PK	17	Finger poke	76	LP
4	FK	PK	58	Struck by own hand	2	20/400
5	HSK	PK	48	Spontaneous	1	20/1000
6	PBK	PK	75	Struck by wooden stick	29	20/167
7	Bacterial keratitis	PK	71	Struck by wooden stick	13	LP
8	HSK	PK	62	Unknown	99	HM/10 cm
9	Corneal endothelial decompensation	PK	42	Spontaneous	126	LP
10	PBK	PK	78	Spontaneous	52	20/500
11	FK	PK	48	Struck by desk	61	20/1000
12	FK	PK	54	Unknown	12	HM/BE
13	FK	PK	45	Struck by rebar	22	20/400
14	FK	PK	12	Struck by book	27	HM/50 cm
15	KCN	PK	19	Struck by phone	34	20/67
16	KCN	PK	23	Struck by basketball	21	20/200
17	FK	PK	49	Struck by shoes	1.5	20/133
18	HSK	PK	60	Struck by wooden stick	84	20/167
19	FK	PK	43	Struck by cabbage	48	FC/BE
20	FK	PK	29	Fall	56	HM/40 cm
21	Interstitial keratitis	PK	53	Punch	84	20/67
22	Corneal perforation	PK	50	Struck by door	84	20/133
23	Interstitial keratitis	PK	53	Punch	84	20/40
24	Ocular chemical injury	LK	46	Struck by cages	204	HM/BE
25	FK	LK	39	Punch	24	20/200
26	KCN	LK	16	Struck by elbow	28	Unknown
27	FK	LK	72	Struck by own hand	72	20/50
28	Interstitial keratitis	LK	47	Unknown	11	HM/30 cm
29	KCN	LK	48	Unknown	5	20/400
30	KCN	LK	16	Spontaneous	31	20/80
31	KCN	LK	20	Unknown	12	20/67

PBK, pseudophakic bullous keratopathy; HSK, herpes simplex virus; KCN, keratoconus; FK, fungal keratitis; PK, penetrating keratoplasty; LK, lamellar keratoplasty; HM, hand moving; FC, finger counting; BE, before eyes; LP, light perception.
